# Grounding Wellness: Coloniality, Placeism, Land, and a Critique of “Social” Determinants of Indigenous Mental Health in the Canadian Context

**DOI:** 10.3390/ijerph20054319

**Published:** 2023-02-28

**Authors:** Viviane Josewski, Sarah de Leeuw, Margo Greenwood

**Affiliations:** 1 School of Nursing, University of Northern British Columbia, 3333 University Way, Prince George, BC V2N 4Z9, Canada; 2 Northern Medical Program, University of Northern British Columbia, 3333 University Way, Prince George, BC V2N 4Z9, Canada; 3 School of Education, University of Northern British Columbia, 3333 University Way, Prince George, BC V2N 4Z9, Canada

**Keywords:** ecology, critical social determinants of health, geography, land, anticolonial, indigenous mental health

## Abstract

Authored by a small team of settler and Indigenous researchers, all of whom are deeply involved in scholarship and activism interrogating ongoing processes of coloniality in lands now known to many as Canada, this paper critically examines “social” and grounded determinants of Indigenous mental health and wellness. After placing ourselves on the grounds from which we write, we begin by providing an overview of the social determinants of health (SDOH), a conceptual framework with deep roots in colonial Canada. Though important in pushing against biomedical framings of Indigenous health and wellness, we argue that the SDOH framework nevertheless risks re-entrenching deeply colonial ways of thinking about and providing health services for Indigenous people: SDOH, we suggest, do not ultimately reckon with ecological, environmental, place-based, or geographic determinants of health in colonial states that continue to occupy stolen land. These theoretical interrogations of SDOH provide an entry point to, first, an overview of Indigenous ways of understanding mental wellness as tethered to ecology and physical geography, and second, a collection of narrative articulations from across British Columbia: these sets of knowledge offer clear and unequivocal evidence, in the form of Indigenous voices and perspectives, about the direct link between land, place, and mental wellness (or a lack thereof). We conclude with suggestions for future research, policy, and health practice actions that move beyond the current SDOH model of Indigenous health to account for and address the grounded, land-based, and ecologically self-determining nature of Indigenous mental health and wellness.

## 1. Grounding Ourselves: A Geo-Eco-Placed Introduction

In Canada, and to some extent, around the world, identity and self-identification are becoming increasingly tricky issues [1,2,3,4]. In late 2021, many Canadians—and certainly everyone involved in health research across the country—watched as the head of Canada’s largest governmental health research funder focused exclusively on Indigenous peoples’ health (the Canadian Institutes for Health Research’s (CIHR) Institute for Indigenous Peoples’ Health) was unceremoniously dismissed from her job, both as an academic and research director with CIHR [5]. This incident was neither the first of its kind nor, by many accounts [6], will it be the last. Increasingly, when employment opportunities encouraging Indigenous applicants are posted, processes are put into place that are meant to confirm Indigenous identities with input from Indigenous communities [6]. With this in mind, “we” are a mixed authorship, two of whom identify as white settlers of European lineage (one of Dutch/Scottish descent, one of German/Canadian) in so-called British Columbia (BC), Canada. One of us identifies as a Cree woman. We co-wrote this paper, for the most part, from either Lheidli T’enneh/Dakelh Territory (Prince George) or Syilx Territory (Okanagan) in so-called BC. We have a combined history of more than 25 years of working together, in various configurations. As individuals and as a team, we are fundamentally oriented to anticolonial practices, research, activism, and writing—by which we mean that we believe structures of coloniality (wherein markers of colonialism such as white supremacy, disavowal of Indigenous ways of knowing and being, racial capitalism, heteropatriarchy, and extractivism are privileged and understood as hegemonic) are antithetical to life and wellness and should thus at every turn be rallied against and dismantled. 

We know. Most academic papers begin with a far-reaching assertion that sets the stage for a series of clearly delineated arguments and logics. Inevitably, the words and sentences in such papers are set into neat and tidy columns, divided out into paragraphs roughly the same size and with anchorage in various citations that demonstrate familiarity with other research, all of which is tidily summarised in a summative but forward-gesturing conclusion. We have written many such papers, including many that have considered issues of physical and mental health, especially as determined by colonialism for Indigenous people in Canada and beyond, but including a focus on other broadly marginalised people and places who live with the grave health inequities meted out to: people in northern, rural, and remote geographies; those who are LGTBQ2S+, experiencing poverty, disabled, and/or deemed fat; those with addictions and/or mental illnesses; the elderly and institutionalised; and/or racialised and immigrant people [7,8,9,10,11,12]. All these people, as much of our writing and research has established, live and have for a long time lived with greater burdens of poor health when compared with other Canadians (because the majority of our research is Canadian-focused) who are settler/white, economically privileged, heterosexual, fit/able-bodied, neurotypical, and/or living in urban geographies. Again, and as much of our writing has evidenced and documented, health inequities and burdens of poor health are deeply driven by social determinants of health (SDOH), including constructions of race and gender, social exclusion, and education [13,14,15,16].

We thus want to begin this paper in a different way, from a different place. And we use the word “place” intentionally. As is well established in the field of geography [17], place is a grounded, material, emotionally resonant, well-known, and deeply cared-for location within the broader, less intimate “space” (for exceptions to this framing, see Massey [18]). Anchored in this conceptualisation of place, we believe it is worth being very specific about the places from which we are writing. We are writing from and within northern and rural so-called British Columbia, specifically on Lheidli T’enneh, Dakelh, and Syilx Okanagan Territories. These are rural and northern geographies: rurality, remoteness, and northernness are not often cited as specific considerations in the mental health literature, which this paper also addresses (for notable exceptions, see Nelson and Wilson [19], Goodwin et al. [20], and Christensen [21]). Bearing all this in mind, we want to begin “deep in the weeds”. “In the weeds” is a place and concept that is quite opposite to the increasingly popular “blue sky” thinking advocated by a growing number of people, especially in business and mass media, who suggest weeds are antithetical to expansive, creative, and important ways of looking at the world (see, for instance, Shah [22]). We take exception in this paper to the pejorative way “weeds” are constructed. Because…oh, the weeds. The minutiae. The tiny. The stomped on. The overlooked. Do these not have a great deal to say in discussions about mental health? We want you to…

Take a deep breath.

Cast your eyes toward the ground.

Toward the land. To the weeds.

Downward to the muck and mud and dirt upon which your feet tread every day. Cast your eyes downward upon that which you walk.

Notice the puddles and the intricacies of soil. Notice the wildlife of weeds springing up between cracks in pavement. Notice patterns and water scars, plants and dust, snails, and the footprints of birds. Tire tracks and windswept leaves.

Indigenous people—who are different from each other around the world—may nevertheless be understood (broadly) as tethered to and rooted in very specific places. In distinct local lands and in grounds and territories of which (and this is no metaphor) they are born and that are born of them. As Linda Tuahawi Smith [23] writes, people who are Indigenous to specific places (lands, ground, territory) have no other homeland to reference, no other territories to which they might consider returning. Indigenous people, though deeply heterogeneous and greatly varied, are in and of place, anchored in a connection to homelands, even if separated from those homelands upon which their kinship and genealogical being stretch back beyond time itself. Associated with this groundedness of Indigeneity is an undeniable truth that many Indigenous people have asserted, namely, that they have an orientation to the world that is fundamentally rooted in land, water, and ecologies. This is what some Indigenous scholars, activists, and knowledge holders call an ecology of knowledge or theology of place [24,25]. This knowledge is what is fueling so much of the “landback” movement in Canada and beyond. Landback is not a turn of phrase or metaphoric reference. Landback is a literal, material, ground-bound statement, and it is inseparable from the bodies (the human beings, the people) who are calling for it. Landback. Landback. Landback is about health and wellbeing. Landback is embodied. Landback is felt, lived, and experienced. Landback is mental and physical and spiritual and emotional survivance and health [26,27]. Landback recognises that deterritorialisation, or the forcible removal of Indigenous people from land and territory, has been fundamental to the Euro-colonial project of nation- and state-building; deterritorialisation has had and continues to have resonant and devastating impacts on Indigenous mental health and wellness [28,29], which is why it is important for you to take a moment and look toward the earth. To territory. To territory, which might be stolen and occupied. Toward the ground. Toward the small, the all-too-often overlooked.

Indigenous ways of knowing and being are not the only reason to cast your eyes soil-ward. Many non-Indigenous settlers living in Canada, such as Black people and People of Colour newcomers (who are racialised in ways that lead to different marginalisations) and white settlers of European ancestry, are also coming to understand that the lands now called Canada by so many are, fundamentally, lands saturated in colonial violence and violated by extractive capitalism [30]. This is a truism that many Indigenous peoples and communities have asserted for decades. People from coast to coast to coast across so-called Canada are also and increasingly documenting ways that rural, remote, and especially northern geographies—along with places more broadly falling outside of the 100-kilometre radius of the United States/Canada border where 90% of inhabitants of Canada now live—are experiencing poorer health outcomes as compared with our urban metropolitan neighbours [31,32]. In other words, the specificity of places, including local lands, and the muck, the weeds, and the unique ecologies, matter.

Furthermore, in Canada, the lands upon which we are all standing are connected to lands that cradle the graves of thousands of Indigenous children [33,34,35]. These graves are only now being unearthed, although stories of the graves have been held for generations by Indigenous families and communities. The earth upon which the nation-state of Canada is overlaid, the earth and grounds and lands of British Columbia (BC) and every other territory, holds the bodies of children, the bodies of babies, who perished in the clutches of institutions designed to eliminate them [35,36,37].

And now, with all this mind, we respectfully ask you, before you read anything else in this paper, to breathe deeply and look groundward; the land holds teachings about how settler colonial violence has operated for far too long in what is now called Canada. The land and the environment hold insights into mental wellness (and illness) of Indigenous people across Turtle Island and beyond. Colonialism, and its close relation, coloniality, form structural and systemic foundations that underpin Indigenous mental health. Understanding those foundations allows us all to envision ways of dismantling them, ways to do better, ways to enact radical anticolonial ways of being that support, nurture, celebrate, and lift up new and radical ways of thinking about Indigenous mental health as grounded and located in place, on the land.

That is the work of this paper. To conceptually ground considerations of Indigenous mental health in so-called Canada. To actively link environment (including ecology, land, geography, and the ground) to Indigenous mental wellness. To consider place-based biases. To do this, we are, theoretically and methodologically, guided by the organic and messy nature of land itself. Rather than employing straightforward or standardised methodological approaches to health research, this paper utilises a creative and narrative approach to presenting and discussing anticolonial ideas.

We begin with a critical engagement of the “social” as privileged within determinants of health literatures; though important in pushing against biomedical framings of Indigenous health and wellness, we argue that the SDOH framework nevertheless risks re-entrenching deeply colonial ways of thinking about and providing health services for Indigenous people. SDOH frameworks, we suggest, do not ultimately reckon with ecological, environmental, place-based, or geographic determinants of health in colonial states that continue to occupy stolen land. These theoretical interrogations of SDOH provide an entry point, first, into an overview of Indigenous ways of understanding mental wellness as tethered to ecology and physical geography, and second, into a collection of Indigenous narrative articulations from across what is now called British Columbia. The narratives presented in this paper are derived from a critical ethnographic study informed by Indigenous and anticolonial perspectives, entitled Towards Cultural Safety in Mental Health and Addictions Contracting for Urban Indigenous Peoples: Lessons from British Columbia [38]. The research was directed by ethical guidelines developed for research with Indigenous peoples [39] and received ethics approval from the Research Ethics Board at Simon Fraser University that served as the primary affiliation of the principal investigator (Josewski) on the study, as well as the participating community partners, including eight community-based provider organisations (one non-Indigenous and seven Indigenous) and two regional health authorities. The study was designed to explore urban Indigenous providers’ perspectives on mental health and the delivery of addictions services within the context of different funding and contractual arrangements.

After broadly surveying land-based and land-informed healing practices that have proven effective in addressing poor mental health, we weave participant narratives, in the form of Indigenous voices and perspectives, into our critical analysis of the literature to examine why there appears to be a hesitancy about getting dirty, about sinking hands (and feet and hearts and spirts) into the mud and ground when it comes to discussions about Indigenous mental wellness. Ultimately, we return to calls for landback, which we explore as a deeply embodied and material call, and we conclude that the refusal of muck and mess is partly locatable in discourses of sanitisation—especially in medicine—discourses that we extend as metaphors for white Euro-colonial settler ways of knowing and being. As we bring all strands of our critical discussion together to muse about a grounded, dirty, messy, ecologically infused future, we make a series of suggestions for future research, policy, and health practice actions that move beyond the current SDOH model of Indigenous health to account for and address the grounded, land-based, and ecologically self-determining nature of Indigenous mental health and wellness. Considering the clear and unequivocal evidence, in the form of Indigenous voices and perspectives, about the direct link between land, place, and mental wellness (or a lack thereof), place, we conclude, must be recentred in all conversations about determinants of mental health and wellbeing.

## 2. Beyond Social: Determinants of Mental Health and Their Distance from Land, Water, and the Grounded World

Over the last two decades, the social determinants of heath (SDOH) have “ascended” into the realms of research, literature, and policy conversations about the underpinnings of and strategies for improving population mental health and wellbeing, and for reducing rates of mental illness, substance use, and related inequities at the population level (see, for instance, Alegria et al. [40]; Allen et al. [41]; Compton and Shim [42]; Patel et al. [43]; World Health Organization [44]). The SDOH acknowledge the need for an exploration beyond the individualistic, biomedical approaches to mental illness that have long dominated mainstream discussions about mental health and substance use [42]. Through a SDOH lens, mental health is viewed as a multifaceted social, economic, political, historical, and cultural phenomenon and, as such, as an important site where conditions of privilege and disadvantage are (re)produced [45]. In so doing, questions of race, class, and gender are brought into the conversation about (ill) mental health [40,42].

The rise of SDOH frameworks has led to more contextualised analyses of the enduring inequities lived by Indigenous people and children in Canada (see for example, Greenwood and de Leeuw, 2012 [46]; Richmond [47]). Yet, as we contend, the SDOH framework is nevertheless at risk of re-entrenching deeply colonial ways of thinking about and providing health services for Indigenous people. This position, which we will expand on throughout this article, is based on one foundational observation. Primarily concerned with the “social” factors determining the health status of individuals and groups, the existing SDOH literature has tended to omit and/or marginalise determinants that Indigenous people identify as imperative to understanding and alleviating the enduring mental health inequities experienced by Indigenous people and communities in Canada and globally [7,48,49,50].

Grounded in Indigenous epistemologies and relational ontologies, Indigenous models of determinants of health identify Indigenous-specific determinants that go beyond the “social” and emphasise the deep interconnections that exist between the physical, spiritual, emotional, and mental dimensions of health and wellbeing (see for example, Greenwood and de Leeuw [46]; Manitowaabi and Maar [51]; Reading-Loppie and Wien [50].) This includes, among others, a sacred and spiritual connection to land [52,53,54,55,56]. Although Indigenous worldviews are diverse, in many Indigenous cosmologies [57,58,59,60,61], land—a notion that encompasses all elements of the natural world, including water, air, soil, earth, mountains, plants, animals, and ecological non-human organic matter—is at the basis of Indigenous ways of knowing (epistemology) and being in the world (ontology) [60,62,63]. Land, or the natural world, is seen as constitutive of and inseparable from human existence and wellbeing [52], unlike the anthropocentrism embedded in many non-Indigenous Western worldviews, which “regards humans as separate from and superior to nature” [64] (para. 1). The interconnections between land and Indigenous ways of knowing and being are absolutely foundational to Indigenous identity [28,29,57,58,65,66,67] (see also Chapter 6) and are reflected within Indigenous knowledge(s), language(s), pedagogy, art, culture(s), and spirituality [52,53,60,62,68]. In many Indigenous creation stories, land and physical geographies are animate and powerful forces that have given existence to people [69]. This spiritual connection between land and human beings not only grounds Indigenous mental health and wellbeing in specific geographies, places, and ecologies but is itself grounded in a deep respect for land as an autonomous force that exists and matters in its own right—outside the social realm of human experience and being-ness [47,56,61,70,71,72]. The grounded-ness of Indigenous determinants of mental health and wellbeing, as articulated by many Indigenous authors [6,7,47,73,74], has important implications for understanding and responding to the mental health inequities that Indigenous people and communities live. These implications are too easily overlooked, however, and cannot be fully understood because of a privileged place afforded to the “social” in SDOH frameworks [7].

Sublimation of Indigenous place (and by extension land, territory, and ground) has long been a colonial exercise for power, control, and resource extraction. The colonially imposed disconnection of Indigenous people from their lands through the imposition of the reserve system operated in tandem with banning traditional food and medicine harvesting and the refusal, by colonial structures, to recognise existing Indigenous land rights and governance. Displacement and deterritorialisation strategies were essential aspects of colonial and neocolonial projects, now well understood as root causes of many mental health inequities among Indigenous people in Canada [13]. Extending effects of forced displacement is a concurrent lack of understanding about, especially in biomedicine—the dominant Western model of health care—the health benefits of emplacement and reterritorialising, about land and ground and dirt and ecology also being places of healing, resistance, and decolonisation [72,75,76]. Indigenous communities are increasingly using land-based and land-informed forms of healing to successfully promote individual and community mental wellness and resilience [76,77,78,79,80]. These forms of healing push back against medical colonialism and anti-Indigenous racism that continue to constrain Indigenous people’s equitable access to culturally safe and effective mental health and addictions care [38,81].

Before colonisation, Indigenous people enjoyed good mental health, supported by traditional medicine and healing systems and deep connections to family, community, and land, all of which formed an integral part of political, economic, social, cultural, and spiritual life in Indigenous societies [51]. The knowledge arising from land and ecology and place was passed down from generation to generation, ensuring healthy and vibrant Indigenous communities across Turtle Island. Colonial legislation and policies severely disrupted these relationships and the intergenerational transfer of knowledge, which had profound consequences for the mental health and wellbeing of future Indigenous generations [82,83,84,85,86].

Colonialism—both in its historical and contemporary forms—is often considered to be the most foundational determinant of ill mental health and related inequities for Indigenous populations [13,17,46]. Colonisation imposed complex systems of control over Indigenous societies through legitimated or “legal” processes of dispossession, displacement, and confinement, which disconnected Indigenous people from their ancestral territories, kinship, cultures, and knowledge systems, undermining their ability to pursue healthy and fulfilling lives [28,29,57,58,65,66,67,86]. The historic and land-based trauma associated with the imposition of the reserve system and, in particular, residential schools—the assimilationist agenda behind which was to “kill the Indian in the child” [35] (p. 130)—has had lasting and damaging effects on Indigenous identities and mental health and wellbeing. Both the reserve system and the residential school system produced painful ruptures in families and communities while simultaneously disrupting Indigenous people’s physical, mental, emotional, and spiritual connections to the land [13,87,88,89,90,91,92,93]. Compounding these impacts were multiple traumas stemming from other colonial policies and practices, such as the outlawing of Indigenous cultural practices, the Sixties Scoop, and present-day child welfare interventions, which continue to remove Indigenous children from their families and communities at rates much higher than for any other group [94,95,96].

The mental health inequities lived by Indigenous people in Canada are a direct reflection of these colonial realities [13,17]. Across Canada, many Indigenous communities are burdened with high rates of substance use [97]. Depression [98], suicide [99] and especially youth suicide [86], and violence [100] are also prevalent. Today, it is widely recognised that these issues are closely linked with historic and intergenerational traumas associated with residential schooling, the extensive loss of culture, language, land, and knowledge, and the consequent erosion of Indigenous cultural identities [35,98,101,102,103,104,105,106].

Despite the importance of place, land, environment, family, togetherness, culture, and identity to Indigenous health and wellbeing, these elements are often missing from Western biomedical approaches to healing [107] (p. 25). Indeed, in many ways, biomedicine remains a site of profound colonial power. As Allan and Smylie [13] point out, the individualistic and bio-psychological understandings of mental illness privileged by the dominant biomedical model of mental health prop up deep-seated racist stereotypes about Indigenous people by pathologising that which are socially constructed problems. What is (incorrectly) inferred in too many discussions about Indigenous mental health is that the root causes of mental health inequities faced by Indigenous people are located within the Indigenous psyche, culture, and/or biology (for example, poor genetics) [13,108,109,110]. Indeed, discourses about the ‘disordered Aboriginal’ (whether this refers to the ‘alcoholic’, ‘depressed’, or ‘traumatised’ Aboriginal) have largely been a product of a colonial mentality and its racialising and culturalist assumptions that find expression in a “tendency to embrace panoptic explanations which uncritically attribute psychopathology to broad historical and cultural phenomena while ignoring local cultural heterogeneity, continuity, and adaptation” [110] (p. 198). The resulting deficit-based discourse is fueled by population health statistics and media portrayals depicting Indigenous people as sick and/or dysfunctional, and therefore incapable of autonomous decision making [38,111,112,113,114]. This further perpetuates and entrenches longstanding racist and paternalistic Indigenous–settler relations within the health care system and beyond [81,115,116,117]. Racist images of Indigenous people and culture(s) as dirty, backwards, superstitious, and inherently sick all serve to legitimise assimilationist, paternalistic, and discriminatory policies and practices, including the creation of racially segregated Indian hospitals in which Indigenous patients—in some cases, children—were subjected to experimental surgeries and inhumane treatments, such as forced sterilisation [115,116,117]. That these ideas remain prevalent and influential within the health care system today is evidenced in the ways anti-Indigenous racism continues to shape Indigenous people’s experiences of and opportunities to access culturally safe and relevant health care (see, for example: Tang and Browne [118]; Browne et al. [119]; Browne et al. [120]). The continued legacy of epistemic racism reflected in the marginalisation of Indigenous ways of knowing and practising in the Canadian (mental) health care system vis-à-vis the dominance of Western biomedical knowledge is just one manifestation of this ongoing colonial reality. As a result, many Indigenous people choose not to, or are hesitant to access, mainstream (Western) mental health and addictions services and supports [121]. Instead, they call for more holistic approaches to mental health and wellbeing that are grounded in Indigenous knowledges and healing practices that strengthen community resilience and address challenges arising from a continuing legacy of colonisation and the disconnection from land [73,82,106,122,123,124]. In response to these demands, the Truth and Reconciliation Commission of Canada (TRC) “call[ed] upon those who can effect change within the Canadian health care system to recognise the value of Aboriginal healing practices and use them in the treatment of Aboriginal patients in collaboration with Aboriginal healers and Elders where requested by Aboriginal patients” [35] (p. 3). The significance of these calls is underscored by an emerging Indigenous and non-Indigenous scholarship demonstrating the ways in which community-based healing initiatives that restore Indigenous knowledge(s) and traditional medicine can facilitate mental health and healing and provide a pathway to ameliorating existing mental health inequities [78,79,80,82,125,126,127]. In the next section, we turn to an exploration of land-based healing as a critical Indigenous-led decolonising approach to enhancing individual and community mental health and wellness [80,82,128] that “has largely remained undefined within mainstream mental health promotion and intervention” [80] (p. 90).

## 3. A Broad Survey of Current Work about Land-Based and Land-Informed Healing

If a central aspect of colonialism is disconnection from place, community, culture, land, ecology, and spirituality, then conversely, decolonialisation and healing must centre and focus upon restoring a sense of connection with community, place, culture, land, ecology, and spirituality [68,80,129,130]. Though relatively small, the body of research and literature on land-based and land-informed healing initiatives is rapidly growing [108,131,132]. Improved individual and community mental health and resilience outcomes have been demonstrated in Indigenous communities [79,107,131,132,133,134], especially among children and youth [135,136,137,138,139]. Analysis of three years of Indigenous mental health and wellness data, including data from the National Native Alcohol Drug Abuse Program (NNADAP) and the National Youth Substance Abuse Program (NYSAP), shows that land-based specific cultural interventions across Canada improved individual mental wellness from 4% to 18% [133]. Indigenous-led land-based treatment and healing initiatives have emerged across many Indigenous communities around the world, including Canada [56,68,78,80,108,132,140], as organisations have galvanised efforts to rekindle connections to the land for “the reclamation of spiritual, physical and psychological health and for the restoration of communities characterised by peace and harmony and strength” [129] (p. 44). Though land-based and land-informed healing practices may be implemented alongside Western models of health care, they are uniquely rooted in Indigenous culture, traditional knowledge, spiritual values, ceremonial practice, and intergenerational knowledge transfer [68,80,141]; they are also highly localised. The myriad approaches to land-based and land-informed healing include stories, creative arts, cultural and ceremonial practices, traditional arts, crafts and foods, land skills education, and activities.

Storytelling has long been used to transmit traditional knowledge between generations and to foster and reinforce Indigenous identity, culture, language, and connection to the land [54]. Stories are grounded in very specific local knowledge of a region’s natural history, coupled with complex layers of family and personal experiences and a deep connection to the past, and therefore to Indigenous identity [142]. Innovative storytelling projects can thus help to “engender interconnectiveness and interdependability through their cultural, scientific and ecological teachings” [143] (p. 14). Especially for children and youth who do not grow up on their ancestral landscapes, storytelling offers a powerful teaching tool that enables Indigenous children to learn about the importance that land has for their culture, identity, and wellbeing [144]. These connections can be cultivated further through the use of creative arts, which are increasingly recognised as offering complementary ways for children and youth to explore and express relationships between land, identity, and culture [145,146].

Indigenous storytelling and creative arts projects are examples of land-informed healing practices that may or may not take place on the land. Land-informed healing extends and reflects the belief that Indigenous language(s), knowledge(s), and culture(s) originate from the land [80,147], and that landscapes themselves “house” stories [148]. According to Okanagan scholar Jeanette Armstrong, “all Indigenous people’s languages are generated by a ‘precise geography’, which informs how the world is viewed, approached and expressed verbally by its speakers through stories” (Armstrong as cited in Mussi [149] (p. 8)). Indeed, as Anishnaabe-Kwe (Ojibway) scholar Cheryle Partridge [150] reminds us, in many Indigenous origin stories, human beings sprang from the land, or Mother Earth, and it is this relationship and caring for this relationship to the land that is at the centre of many Indigenous stories, ceremonies, teachings, prayers, and songs.

Indigenous land-based healing integrates Indigenous ways of knowing, doing, and being, while honouring the spiritual, ancestral, and physical relationships between land, Indigenous identity, and culture. In “The land is a healer”, Dene scholar Redvers [80] describes the land’s central role in promoting mental health and resilience in the way that it uses what knowledge holders describe as the “experiential language” of the land to help people remember and feel the ancestral and spiritual connectedness they have to all living things, including earth, fire, water, and air.

This same concept is beautifully illustrated by Stevenson [151], who shares a Métis account of the healing power that can be found in witnessing the sunrise. In “Waniska” (Wake Up!), “being present during this time” she writes, “will stir and mirror what is naturally awakening inside you” (p. 15).

*Being with nature…helps make sense of things. Sitting quietly with our Other-Than-Human Kin fosters the quiet mind. The Still Place where you can hear the voice of nature or instinct as it stirs in your internal world. Gratefulness humbly offered to the Other-Than-Human world helps to restore the bond between humans and nature. As that bond strengthens, you can no longer ignore the fact that you are connected to everything else. You begin to see the Sacred in everything.*

[151] (p. 14)

Ceremony or spiritual practices on the land help to renew a sense of connectedness and harmonious balance between the self and the cosmology of the land—a process entwined with “love” for all kin, human and Other-Than-Human, and a recognition of the sacredness of all life [151]. In this way, land instills a sense of meaning, purpose, belonging, and hope, all of which strengthen mental wellness and resilience, as Elder Jim Dumont describes in the First Nations Mental Wellness Continuum Framework [152]. As he writes, mental wellness requires a balance of one’s spirit, heart/emotions, mind, and body.

*This balance and interconnectedness is enriched as individuals have purpose in their daily lives, whether it is through education, employment, and caregiving activities or through cultural ways of being and doing; hope for their future and those of their families that is grounded in a sense of identity, unique Indigenous values, and having a belief in spirit; a sense of belonging and connectedness within their families and to community and culture; and finally a sense of meaning and an understanding of how their lives and those of their families and communities are part of creation and a rich history.*

[153] ((p. 4), authors’ emphasis)

As Aikenhead and Michell [154] explain, Indigenous knowledge(s), including healing knowledge, originate in observations, interactions, and experiences of living with the land. Although Indigenous knowledge(s), cultural, and healing practices are land-specific and differ among First Nations, Inuit, and Métis, there are many commonalities, including the importance of ensuring a reciprocal relationship with the land for maintaining equilibrium of the universe, and by association, physical, mental, emotional, and spiritual health and wellbeing. Since time immemorial, it has been the role of Knowledge Keepers, Elders, and Healers to share this knowledge through storytelling, cultural teachings, and ceremonies. Elders have built relationships with the land (and its inhabitants) over time, are keepers of traditional and cultural knowledge, and therefore, play a key role in intergenerational knowledge transfer (the passing of knowledge and experience of social, cultural and ecological contexts to the next generation) [155]. Elders thus play an important role in in the development and delivery of land-informed and land-based healing initiatives [152,153,156,157]. Many land-based healing initiatives include “Bush schools” in which Elders teach land-based activities and skills (e.g., harvesting traditional foods, planting medicines, fishing, hunting, and trapping), while sharing cultural teachings, stories, and spiritual practices about the interdependencies of plants, animals, rocks, water, and human kin, and the ethical responsibilities that flow from this interconnectedness, such as giving thanks and respecting and caring for all living things. Many such programs tend to focus on younger generations and families and have the potential to strengthen social and intergenerational bonds [107,132,158].

Two successful examples of land-based healing programs developed by and for Inuit youth and communities include the Aullak, Sangilivallianginnatuk (Going Off, Growing Strong) program and the Makimautiksat Youth Camp. The Aullak, Sangilivallianginnatuk program in Nain, Nunatsiavut aims to promote mental wellness and resilience of Inuit youth struggling with suicidal thoughts and behaviours through traditional Inuit land-based activities [135]. The program connects youth with harvester-mentors from the community, engaging them in such land-based activities as building Kamutiks (sleds), hunting, trapping, fishing, and gathering of wood and plants that are then delivered to Elders and community members. Evaluation of the Aullak, Sangilivallianginnatuk program demonstrated a noticeable decline in suicide attempts and rates among the youth and the community in general, with participating youth reporting having gained Inuit-specific traditional skills and self-confidence as a result of the program [135]. In Nunavut, the Makimautiksat Youth Camp fosters mental health and wellness among children and youth through a curriculum focused on promoting Inuit Qaujimajatuqangit (Inuit traditional knowledge) and Inuit-specific traditional skills, including a two- to three-day on-the-land camp delivered by Elders and other Knowledge Keepers [159]. The on-the-land component includes hands-on activities such as “catching, cleaning, and preparing dry fish, setting up a camp, camp safety, learning about wildlife, the land, and our relationship with our environment” [159] (p. 9). In one report, children and youth from the six participating communities said that after the camp, they felt “more happy, cheerful, and energetic” and experienced “a decrease in feeling sad and feeling miserable” [159] (p. 4). These positive outcomes were reaffirmed by the parents who “reported seeing significant positive behaviour and attitude changes in the children” as well as strengthened relationships and Inuit knowledge among youth and the community at large [159] (p. 4).

These findings draw attention to the indivisibility of the determinants of Indigenous mental health. Strong interconnections exist between land, place, ecology, Indigenous self-determination, language, culture, stories, traditional foods, identity, family, and community. These are valuable strategies and approaches with the potential to restore and strengthen mental health and healing from past and ongoing forms of colonial trauma. By the nurturing of intergenerational and family connections and developing a shared sense of belonging and empowerment, land-based healing strategies can play a key role in healing from the effects of historical and intergenerational trauma associated with such policies as residential schools and child welfare practices [68,76]. Moreover, because land-based healing programs emphasise relational and local place- and culture-based ways of knowing and learning (rather than approaches based on pathology), such projects tend to be Indigenous-led and community-driven, building community strengths and opportunities for Indigenous self-determination. Compelling evidence demonstrates that self-determination and increased cultural continuity are important factors in protecting against and reducing the occurrence of suicide [160,161].

A closely related expression of the interconnections between land, mental health and healing, culture, and self-determination is the growing focus on traditional foods, Indigenous food sovereignty, and land stewardship. Traditional foods-based initiatives, such as berry picking, salmon harvesting, hunting, trapping, gardening, and foraging provide a strong platform for enhancing both individual and community mental health and wellness by strengthening Indigenous food sovereignty and fostering the connection between a community, its landscape, and cultural roots while positively impacting the health of local ecosystems [162]. A series of initiatives in Australia have been designed to promote Indigenous-led, community-driven participation in activities on the land with the aim of promoting ecological, spiritual, and human health. These initiatives are also known as “Caring-for-Country” projects (see Kingsley et al. [163] and Berry et al. [164], for examples). Such projects can achieve linked goals of mental health and sustainability through: supporting the recognition of the interdependent relationships of humans to all creatures; promoting dignity, identity, and self-determination; and building community strength and opportunities for sustainable economic development, physical activity, and improved diet [162,164,165].

Indigenous land-based and land-informed healing and wellness thus integrates Indigenous ways of knowing, doing, and being. As Redvers [80] emphasises, “one must first experience it directly through practical, culturally-rooted activities, languages, and interactions that return us to the land physically, emotionally, mentally, and spiritually” (p. 102). This focus on experiential learning, often quite literally by getting one’s hands dirty, reflects a core aspect of Indigenous land-based healing [76]—a focus that is absent in mainstream models of mental healthcare, which tend to view healing from a clinical, biological perspective. Moreover, land-based and land-informed healing embodies a deeper understanding of land as more than a material construct of place, where “land is spiritual, emotional, and relational” (Styres as cited in Ferguson [166] (p. 118)). Thus, unlike the individualistic orientation of most biomedical models of mental health care, land-based and land-informed healing practices are oriented to heal relationships, including connections to oneself, family, community, nature, place, and culture. For many Indigenous people and communities, land is a place and source of healing, spiritual regeneration, and resurgences against ongoing settler colonialism [129,132,167].

## 4. Why the Aversion to Dirt? Mental Health, Ecosystems, and the Environment Challenges

Most mental health services continue to be designed and delivered within the dominant paradigm of the psy-disciplines (i.e., psychology, psychotherapy, and psychiatry). Rooted in a biomedical and individualistic framing of mental health, Western models of mental health care fail to substantiate the contextual factors shaping people’s mental health and healing, especially human–nature relationships and the significant role of land in promoting the mental health and wellness of Indigenous people and communities. These models thus remain reluctant to fully recognise and fund land-based and land-informed approaches to mental health and wellness. Even when Western models consider the impact of environment on mental health, such considerations tend to be limited to the social [168] and/or the negative—the environment is seen as hazardous rather than healing and nurturing [156].

As a result, mainstream mental health and substance use treatments routinely seek to isolate patients from their social, physical, and spiritual environments [168]. In Canada, the use of involuntary detainment and treatment, physical restraints, and seclusion remain common practice in mental health [169,170]. After reforms away from institutionalisation, there has been a recent trending toward re-institutionalising the “mentally ill”, fueled by outcries about the opiate crisis [171]. Such practices and service delivery models run counter to Indigenous healing, with the land as a practice and knowledge system. Many Indigenous and non-Indigenous advocates and scholars have argued that such institutionalising practices, which disproportionately impact racialised and Indigenous communities, constitute human rights abuses [169,170,172] and exemplify the ways in which biomedical colonialism and anti-Indigenous racism manifest institutionally within mental health care systems [171].

Moreover, these foundational power structures often remain unchallenged. As Allen et al. [173] observe, “Western medicine and knowledge systems often remain the standard for comparison, for ethical guidelines and for making claims of efficacy” (p. E209). Although there have been widespread commitments to cultural safety and a growing number of attempts to integrate Indigenous healing practices into existing mental health services, the normative hegemony of the biomedical model of mental health, compounded by epistemic racism, means that current funding arrangements for Indigenous community-based mental health services—including land-based and land-informed healing—too often create unique structural barriers for Indigenous organisations [38]. The lack of adequate and sustained funding for Indigenous land-based and land-informed healing initiatives and inequitable compensation for Elders and Knowledge Keepers, identified both by community members and in the literature, is a manifestation of this neocolonial reality [38,68,174].

The enduring hegemony of biomedicalism, despite commitments to the contrary, is illustrated in the following narrative by an Indigenous director of a community-based organisation in what is now known as British Columbia and participant of the *Towards Cultural safety in Mental Health and Addictions Contracting for Urban Indigenous Peoples Research Project* [38]:

*The way that we wanted to provide service here was not to just put a brown face on a mainstream program we wanted to ensure that there was a cultural lens through which our work was done that we had all kinds of pieces around tradition and ceremony and spirituality … as a way of healing and … providing services. … And that hasn’t happened and so … in my mind, there [remains] … a lot of work that needs to be done around that because the need is there.*

[38] (p. 202)

The participant’s account highlights how the dominance of biomedical norms embedded in funding arrangements thwarted the community’s vision of implementing an Indigenous-specific child and youth mental health program and continued to reinforce the dominant culture’s approach to mental health care programing. Adding a different perspective, another Indigenous research participant, who had worked in government and been involved in the funding of urban Indigenous organisations in the area of mental health, shared:

*It was challenging all around for community services because the hospital is the king of health services and has the most money and has the most prestige and therefore the most power so community health services are the poor step child of the hospital system and the community service providers are the poor step children of the community health services. And the Aboriginal community services are, you know, those wicked stepchildren that you hide in the corner. You know, so it, it’s hierarchy again.*

[38] (p. 112)

As Rogers et al. [174] observe, Indigenous health practitioners must constantly negotiate both the Western and Indigenous worlds, with many practitioners expressing frustration with having to constantly defend the credibility and legitimacy of Indigenous ways of healing. As commented by the above Indigenous research participant, “Mental health services told me frankly ‘oh well you’re just paying for crafts’” [38] (p. 107). As Browne et al. [119] observe, funding structures and reporting requirements are often “organised in ways that oblige communities to ‘streamline’ their services to fit conventional notions of essential health services—often narrowly defined in accordance with biomedical conceptualisations of what counts as legitimate” (pp. 305–306). Many mental health-specific funding sources do not consider land-based healing initiatives as mental health programs, and thus, they are not accessible to communities [68]. Even when some program funding has been secured, many associated costs, such as materials needed for cultural rituals and ceremonies, food, and remuneration for Elders, are viewed as illegitimate [38,68].

A decolonised approach to funding requires careful reflection on how colonial relations in Indigenous mental health care continue to be, often unwittingly, re-enacted as a result of taken-for-granted assumptions embedded within, and bolstered by, health care funding institutions. In the dominant Western view, mental health care and healing is a rational science that requires a rational agent (i.e., mental health practitioner/expert) whose practice is based in scientifically produced knowledge/evidence [175]. A human-centric orientation is deeply embedded in biomedical approaches to mental health. This is an orientation fundamentally at odds with Indigenous healing knowledges, which view the land as a Teacher and Healer [80]. The concept of evidence-based mental health practice, as it is currently performed, reflects this colonial bias toward rationality. Although evidence-based practices ideally integrate all forms of evidence [176], current approaches to evidence-based practice privilege knowledge derived through Western scientific methods, for which randomised controlled trials remain the gold standard over knowledge developed through Indigenous research methods [174,177], which emphasise experiential learning [58,178], including land-informed and land-based learning. In the words of another Indigenous study participant:

*All evaluation and evidence-based practices [are] very Western and it’s the same thing with contracting deadlines, deliverables it’s all pushed down through the institution, you know. Everybody is supposed to meet the same boxes. And that doesn’t really work for us so we have to learn how to be flexible but we have to have some kind of evaluation to show that we did something and that it’s at least somewhat valid or we don’t get credibility in the Western culture or the health authority*.

[38] (p. 186)

The dominance of biomedicine within mental health coupled with the normative status of Western ways of knowing (e.g., experimentally derived evidence) stand in the way of seeing Indigenous healing practices and knowledge systems as legitimate and credible. Whereas within a Western paradigm, claims about the efficacy of a therapeutic intervention are based on scientific evidence (knowledge derived using the scientific method), the efficacy of Indigenous healing is often grounded in and intrinsic to local specificity, knowledges, and relationships, including geographic landscapes, placed-based knowledge, and ancestral and spiritual relationships.

A commonly cited barrier for more sustained support for land-based healing is a lack of evidence showing the benefits of traditional land-based or land-informed healing interventions on health and wellbeing (see, for example, Ahmed et al. [179]). Though some evidence in support of land-based healing practices exists, such evidence is often located at the bottom of the so-called hierarchy of evidence, because it tends to be derived from single case studies rather than from experimental study designs and is missing considerations of economic or cost analyses. Furthermore, it tends to be based on subjective self-reported outcome measures as opposed to objective measures. Whereas the curative treatment goal of most biomedical and psychotherapeutic interventions, often measured in the absence of dysfunctional or pathological symptoms, allows for a relatively easy assessment of efficacy, the restorative and transformative nature of much Indigenous healing creates challenges for or may even preclude objective assessment [110]. From a Western positivist stance, these limit the generalisability, scientific rigour, and applicability of the evidence produced [180]; and yet, evidence-based practice primarily defined through a Western lens limits the ability to understand and address the specific concerns, priorities, and contexts of Indigenous people and communities [57,181,182].

What is more, “transformative healing involves a continuous, although not necessarily smooth, developmental process in which the patient undergoes changes in physical, behavioral, cognitive, emotional, social, spiritual, and/or existential functioning” [110] (p. 193)—yet, how does one objectively observe, measure, and quantify spiritual growth? Land-based healing thus muddies the crystal-clear waters of dominant Western notions of what counts as evidence. As one Elder, who sat on the board of directors for one urban Indigenous organisation, observed, the question of what constitutes strong evidence of a program’s effectiveness is “not black and white”; rather, Indigenous land-based and land-inform healing systems raise deeper epistemological and methodological questions: “how do you measure success?” and perhaps even more importantly, “if you can’t measure that success in your service delivery according to their standards, then is it a failure?” [38] (p. 186). The radically different ontological and epistemological assumptions underpinning Indigenous land-based approaches to mental health and healing compared to Western biomedical models of mental health mean that Indigenous land-based and land-informed methodologies do not fit within the positivist paradigm [174]. Evidence produced outside the Western knowledge framework, and especially evidence derived from Indigenous research methods, such as experiential learning, storytelling, and learning from Elders, is considered weak, inconclusive, or ideological [183], and is thus often marginalised or dismissed altogether [38,174].

Colonial and racist stereotypes that cast Indigenous cultures as primitive and dirty perpetuate a deep-seated mistrust toward Indigenous healing systems [81]. As Kelm [184] notes, Western knowledge systems played, and still play, a key role in portraying Indigenous forms of medicine as “quackery or superstition” (p. 153) and consequently in its rejection as “unscientific” [185] (p. 5). With its connection to dirt, muck, and spirituality, the notion of land-based healing is seen as antithetical to the beliefs, norms, and values upon which the presumed superiority of Western science and philosophy of biomedicine rests. Springing from this juxtaposition of modern/traditional and Western/non-Western is a deep aversion to dirt within Western culture, and by extension, land-based forms of healing. This aversion reverberates across all psy-disciplines and is encapsulated in both Western science and biomedicine by the idealisation of whiteness. White symbolises truth, cleanliness, purity, and ethical/moral supremacy. One of the best-known symbols representing modern sciences and medical authority is the white coat; according to a recent article, 97% of medical schools in the US have a white coat ceremony [186]. Against this backdrop, anything outside of whiteness, including People of Colour and Indigenous people and their healing systems—especially those practices that quite literally may involve dirt—becomes suspect, filthy, unscientific, and potentially corrupt. The unwillingness to provide adequate and sustainable funding to Indigenous-led land-based healing initiatives, including Elders, Spiritual Advisors, and Healers, therefore epitomises the continued embeddedness of anti-Indigenous racism and biomedical colonialism within Western mental health care.

Other neocolonial processes enacted at the system-level that create barriers to land-based forms of healing bring into focus issues of jurisdiction and equity. The federal and provincial/territorial governments have yet to clearly define areas of responsibility over Indigenous people’s health, resulting in many urban Indigenous people’s even more limited access to land-based and land-informed healing initiatives than those living in First Nations communities and Inuit territory. Due to federal funding restrictions, many Indigenous-specific substance use and mental health programs that include land-based healing components are only available to status First Nations and people living on-reserve—a consequence of the enduring colonial mentality that places Indigenous people and cultures into rural and remote areas, even though more than half of the Indigenous population in Canada lives in urban centres [187]. Jurisdictional boundaries that correspond to health authority regions can further compound issues of access and availability of land-based healing for urban Indigenous people where access to land is often limited. In her work with urban Indigenous community-based organisations in British Columbia, Josewski [38] describes how one organisation was denied funding for the implementation of a community-led land-based healing initiative simply because the land in question was located outside the regional boundaries of the local health authority. As these findings illustrate, though Indigenous knowledge(s) and ways of knowing are beginning to transform how mental health and healing are understood and approached, colonial and other racist discourses and structures perpetuate policies and practices that, inadvertently or not, deny many Indigenous people access to culturally safe and grounded mental health services and supports.

## 5. Braiding the Mess: Thinking about How to Include Land, Water, and the Grounded World in Terrains of Indigenous Mental Wellness

We are in no way interested in romanticising Indigenous ways of knowing and being or, conversely, in demonising Western ways of knowing and being. Indeed, we celebrate that Western scholarship is increasingly attentive to spirituality and the impact of environmental degradation and climate change on mental health [156]. Instead, our intent is to unsettle taken-for-granted assumptions about the ungrounded placelessness of many social determinants of health frameworks—by highlighting the power of land-based and land-informed healing for promoting the mental health and wellbeing of Indigenous people, especially children and youth, families, and communities. We believe it is precisely through such unsettling work that progress might be made toward dismantling the dominance of biomedicalism. Social determinants of health were (and are) meant to push against the contours of a pure biomedicalised system, which unto itself is a settler-colonial manifestation within the mental health care system. Specifically, our argument reflects a moral concern with the ways in which the ongoing dominance of biomedicalism constrains opportunities for mental health and healing, self-determination, and, more broadly, social justice and equity for Indigenous people.

Holistic determinants of health models, such as the web of being [188], emphasise the interweaving of the mental with the physical, social/emotional, and spiritual dimensions of health and healing, and the importance of land as an interrelated key determinant of Indigenous health. Unlike the individualistic orientation of most biomedical models of mental health care, land-based healing practices are therefore oriented toward healing relationships. This includes “All of Our Relations”—human kin and the Other-Than-Human kin, which are “the other inhabitants of this great land—plants, animals, water, rocks, everything above and below the surface of Mother Earth” [150] (p. 7). Understanding these interconnections between humans and the natural world is central to understanding Indigenous approaches to mental health and healing [73,80,189], as well as the difference between looking at the land as a relation rather than a resource [129]. Indigenous land-based and land-informed healing programs in Canada are founded in the unique spiritual and ancestral relationships that First Nations, Inuit, and Métis people have with the land. Land-based or land-informed healing is about restoring and strengthening these bonds by learning from, with, and about the land [190,191]. The interrelated pathways by which land-based and land-informed healing promotes Indigenous mental health and wellbeing are discussed within the literatures on the determinants of Indigenous health, highlighting the importance of self-determination, spirituality, cultural identity, family, culture, and community. For many Indigenous people and communities, land is seen as foundational to individual and community mental health and wellness [91,156,192]; it is a place and source of healing, spiritual regeneration, and resurgences against ongoing settler colonialism [129,132,167].

The dominant model of mental health care has been critiqued for legitimising and enacting human rights violations through medically coercive practices that disproportionately impact racialised and Indigenous communities [171]. Standing in stark contrast to this model are land-informed and land-based forms of healing that are reflective of an Indigenous human rights approach to mental health and wellness and are rooted in an Indigenous determinant of health model. Indigenous people have an inherent right to put this model into practice. In addition to affirming Indigenous people’s rights to mental health and access to traditional medicines and healing practices, Article 25 of the UN Declaration explicitly affirms that:

*Indigenous people have the right to maintain and strengthen their distinctive spiritual and material relationship with the lands, territories, waters and coastal seas and other resources, which they have traditionally owned or otherwise occupied or used, and to uphold their responsibilities to future generations in this regard.*

[193]

The linkages that exist between Indigenous peoples’ individual and collective rights, their mental health, and their relationship to land mean that governments have both an ethical and legal obligation to ensure that all First Nations, Métis, and Inuit children, families, and communities—whether they live in urban, rural, or remote environments, on- or off-reserve—have access to land-based and land-informed healing. Recent developments in relation to Indigenous people’s human rights provide, therefore, an important backdrop to current and future efforts focused on promoting equity in and improving mental health and wellness outcomes for Indigenous people through land-based and land-informed healing. Of particular importance is Canada’s An Act respecting First Nations, Inuit and Métis children, youth and families (2019), which is intent on contributing to the implementation of UNDRIP and ensuring that Indigenous children are able to access their rights [194]. According to the Act, determining “the best interests of an Indigenous child” is, thus, incumbent on considering such factors as “the child’s cultural identity and connections to the language and territory [land] of the Indigenous group, community or people to which the child belongs” (p. 7).

Improving equitable access to land-based and land-informed healing for Indigenous people, families, children, and communities will, however, necessitate transformative change across the structural, system, and service delivery levels [195]. At the structural level, actions are required that will enhance the credibility of land-based and land-informed healing programs [134] and unsettle the longstanding colonial power relations that continue to delegitimise Indigenous approaches to mental health, research, and evidence, with profound consequences for resource allocation and access to land-based and land-informed healing. At the system level, dedicated long-term funding for land-based and land-informed healing initiatives led by Indigenous Elders, Knowledge Keepers, and organisations is needed not only for program accessibility and sustainability, but also to support and strengthen the development and capacity for culture-based and strength-based research and indicators [108,134]. Ensuring that funding is flexible in how and when it can be used supports local Indigenous leadership and community ownership, which is vital to the effective delivery of any health service or program, but especially for land-based and land-informed healing initiatives [108]. These actions would align with the imperatives put forward by UNDRIP [193] and the Truth and Reconciliation Commission [35] and would represent an important step toward decolonisation, reconciliation, and equity in mental health in Canada.

## 6. Conclusions

What needs to be done will be messy work; this work might also include further investigations into how Canadian-based conceptualisations of mental health and territory could be put to work beyond the modern colonial state of Canada. In this way, the Indigenous voices in this paper might extend around the world. It will be work that takes everyone down “into the weeds”. We believe that all questions about Indigenous mental health and wellbeing must return precisely to the mess, to the muck and mire and dirt and mud and land and soil—and the weeds! In this process, we take inspiration and learn from rivers and mountains and grasslands and valleys and lakes. These are the places from which mental health and wellness will arise. Place must be recentred in all conversations about determinants of mental health and wellbeing. We believe place is a generative site from which a foundation for future dialogue about mental wellness between Indigenous and non-Indigenous people can begin to grow.

## Data Availability

Data supporting the reported results can be found in: Josewski, V. (2020). Moving towards cultural safety in mental health and addictions contracting for urban Indigenous Peoples: Lessons from British Columbia. PhD dissertation, Faculty of Health Sciences, Simon Fraser University. https://summit.sfu.ca/item/19947 (accessed on 27 January 2023).
